# Exploring Digital Health Use and Opinions of University Students: Field Survey Study

**DOI:** 10.2196/mhealth.9131

**Published:** 2018-03-15

**Authors:** Ilaria Montagni, Tanguy Cariou, Tiphaine Feuillet, Emmanuel Langlois, Christophe Tzourio

**Affiliations:** ^1^ Team HEALTHY Bordeaux Population Health Research Center (Unité Mixte de Recherche 1219) University of Bordeaux / Institut National de la Santé et de la Recherche Médicale Bordeaux France; ^2^ Science Politique et Sociologie Comparative Centre Emile Durkheim (Unité Mixte de Recherche 5116) University of Bordeaux Bordeaux France

**Keywords:** eHealth, mHealth, students, mobile application, surveys, Internet

## Abstract

**Background:**

During university, students face some potentially serious health risks, and their lifestyle can have a direct effect on health and health behaviors later in life. Concurrently, university students are digital natives having easy access to the internet and new technologies. Digital health interventions offer promising new opportunities for health promotion, disease prevention, and care in this specific population. The description of the current use of and opinions on digital health among university students can inform future digital health strategies and interventions within university settings.

**Objective:**

The aim of this exploratory study was to report on university students’ use and opinions regarding information and communication technologies for health and well-being, taking into account sociodemographic and self-rated general and mental health correlates.

**Methods:**

This field survey was conducted from March to April 2017. An informed consent form and a paper questionnaire were given to students aged 18 to 24 years in 4 university campuses in Bordeaux, France. The survey was formulated in 3 sections: (1) sociodemographic characteristics and self-rated general and mental health, (2) information about the use of digital health, and (3) opinions about digital health. Data were analyzed using descriptive statistics and tests of independence.

**Results:**

A total of 59.8% (303/507 females) students completed the questionnaire. Concerning digital health use, 34.9% (174/498) had at least 1 health app mostly for physical activity (49.4%, 86/174) and general health monitoring (41.4%, 72/174,), but only 3.9% (20/507) of students had a wearable device. Almost all (94.8%, 450/476) had searched for Web-based health-related information at least once in the last 12 months. The most sought health-related topics were nutrition (68.1%, 324/476); pain and illnesses (64.5%, 307/476); and stress, anxiety, or depression (51.1%, 243/476). Although Wikipedia (79.7%, 357/448) and general health websites (349/448, 77.9%) were the most consulted sources, students considered institutional or official websites as the most credible sources (309/335, 92.2%). There were significant differences in digital health use by gender, field, and year of study. No statistically significant association was found between digital health use and self-rated general and mental health status. Concerning opinions on digital health, although 94.1% (475/505) of students estimated that today’s digital health cannot replace traditional health services and medical consultations, 44.6% (207/464) of students declared that this could be possible in the future, provided that digital health interventions are promoted by institutional or official entities.

**Conclusions:**

University students are largely using the internet for health information seeking, but using less mobile health apps and very few wearable devices. Our data suggest that digital health has the potential for improving health and well-being at the university, especially if digital health interventions take into account students’ profiles, interests, and needs.

## Introduction

### Background

University students represent almost two-thirds of all young adults in Organisation for Economic Co-operation and Development (OECD) countries [1]. As potential future leaders, politicians, and managers, their health and well-being is a world-wide public health priority [2]. Although they can be viewed as a privileged healthy population, university students often report poor health conditions. They have relatively high rates of sexually transmitted and inflammatory diseases due to risky sexual practices [3]; they are at risk of chronic diseases due to sedentary behavior [4], problematic alcohol consumption [5], and drug use [6]; and frequently report mental health problems such as stress, anxiety, or depression, which are often due to academic load and homesickness [7].

This important segment of the population has necessarily wide access to modern information devices (eg, mobile phones, computers, and tablets). Known as digital natives or net generation [8], university students are among the highest users of the internet and new technologies not only for educational purposes but also for communication, recreation, and learning in general, including searching for Web-based information [9].

In view of this, university students represent an important target for digital health interventions. Digital health is defined as the general use of information and communication technologies for health [10], where health encompasses any state of complete physical, mental, and social well-being. Digital health is inclusive of both internet- and mobile-based tools (ranging from websites to mobile phone apps) aimed to prevent and treat diseases, as well as to promote health and well-being. The important role of digital health for university students has been largely recognized, and today, universities are increasingly recurring to digital solutions to improve their students’ health. In the past two decades, several digital health interventions have been tested and diffused in different campuses worldwide. These include, for instance, Web-based programs to promote healthy eating and physical activity [11,12], mobile-based tools to reduce tobacco and drug use [13], apps to decrease sexual risk behaviors [14], and both internet- and mobile-based tools to improve university students’ mental health [15,16]. Most studies have been carried out in experimental settings (eg, randomized controlled trials) and Anglo-Saxon university campuses (eg, the United States, Australia).

In parallel, the number of Web pages providing health information is constantly increasing, and the open digital market is becoming overwhelmed with mobile phone apps and wearable devices for health [17]. Although numerous surveys have been conducted to investigate university students’ Web-based health information seeking behavior [18,19], few survey-research studies [20] have assessed and cataloged current use of digital health in university students in a natural noncontrolled setting, not limited to health information seeking, but including also the download and use of mobile apps as well as smart watch ownership, for instance. Furthermore, little research [21] has been conducted to describe in the student population the association of digital health use with gender and self-rated health and specific characteristics such as field and year of study. Understanding how these individual factors influence digital health use could inform the development of acceptable and successful internet- and mobile-based health interventions in the university setting.

In most of the OECD countries, universities and attached students’ health services are asked to propose health strategies and policies to prevent diseases and promote health within their campuses [22]. Investments in digital health are globally on the rise, but public universities are often constrained by human and economic resources. It is then important to understand which digital health interventions should be implemented as a priority, on which topics and by which means (eg, internet- and mobile-based tools).

### Aim of This Study

To help design and implement future digital health strategies and interventions in university campuses, this exploratory study aimed to provide a general overview on patterns of digital health use among university students in France, extending existing research with updated data on Web-based health-related information seeking and related trustworthiness, and on the use of mobile phone apps and wearable devices for health and well-being. The correlation of digital health use with sociodemographic characteristics and self-rated health was also examined.

## Methods

### Study Population and Recruitment

This study was conducted within the framework of the larger ongoing i-Share cohort study (Internet-Based Students Health Research Enterprise), a French nationwide Web-based survey on the health and well-being of university students, whose principal investigators and operational staff are based at the University of Bordeaux. Drawing on some findings of the i-Share survey [23], we were inspired to look further in the issue of digital health use among university students. This specific cross-sectional questionnaire study was then conducted from March to April 2017 as an exploratory study in a new sample of students at the University of Bordeaux.

A paper questionnaire was administered face-to-face by 9 undergraduate trainees (interviewers) who approached their peers in the halls, canteens, courtyards, and study rooms of 4 campuses, each corresponding to a specific field of study (Literature and Social Sciences, Life and Health Sciences, Science and Technology, and Law and Economy). The quota sampling method was used to recruit students according to their gender and field of study : the interviewers had to approach a predefined number of female and male students in each campus to obtain a representative sample of students according to the student registration database of the University of Bordeaux 2016/2017 (see Multimedia Appendix 1). If students consented to participate in the study, the questioning proceeded after the signature of a written informed consent form. If eligible students declined to participate, interviewers asked them why and documented the reasons for refusal. The inclusion criteria were currently studying in 1 of the 4 university campuses in Bordeaux, France; being French-speaking; and being aged 18-24 years. We excluded those aged 25 years and older because, according to the Bologna process ensuring comparability in the standards and quality of higher education qualifications in Europe, the average age of entrants to the university is 18.5 years [24], and the median age students first graduate from university is under 25 years [25].

### Survey Instrument and Ethics

The questionnaire was co-designed by a team of 4 researchers in epidemiology, health communication, health sociology, and mental health, plus 2 public health undergraduate students, following a 5-step collaborative process. According to this methodology [26], the team identified topics of interest (step 1), reviewed relevant existing survey items (step 2) [18,19,27,28], drafted new survey items and adapted existing ones (step 3), tested a first draft version of the questionnaire (step 4), and refined the draft questionnaire providing a final version (step 5).

During steps 1-3, the team checked for the feasibility of the survey, deciding not to include long scales and limiting the length of the entire questionnaire to less than 20 items because it had to demand reasonable time for completion in particular conditions (eg, while attending courses or revising for examinations). The co-design strategy also allowed determining the final 16 health topics of interest for university students. During step 4, a preliminary test phase with 30 students was carried out to verify the coherence of the questions and the easiness to answer. Collected data were not inserted in the final analyses. These 30 students were approached in the different campuses of the University of Bordeaux and asked to sign a consent form stating that their data would not have been included in the final analyses of the project, and that they were contributing to a test phase. At the end of each test questionnaire, interviewers asked students to comment on the length and interest of the questionnaire. When possible, interviewers asked students to comment on each item in detail. This was done by one-fourth (n=5) of the students participating in the test phase. These inputs, in the form of short transcriptions and notes recorded in a separate report, were taken into account when constructing the final version of the questionnaire (step 5).

The final questionnaire was divided into 3 sections:

- Sociodemographic characteristics: gender, month and year of birth, field of study (4 items: Science and Technology, Literature and Social Sciences, Law and Economy, and Life and Health Sciences), year of study (4 items: 1st year, 2nd year, 3rd year, and >3rd year), as well as self-rated general and mental health on a Likert scale (5 items each: very good, good, average, bad, and very bad).
- Questions about use of digital health: participants were asked whether they had a mobile phone (2 items: yes, no), a wearable device (2 items: yes, no), a mobile health app (2 items: yes, no), and, only for those reporting having a mobile health app, its frequency of use (3 items: often, occasionally, never) and name or topic (open-ended item). On the basis of a list, participants were asked about health topics they had searched for on the internet in the last 12 months (15 items: sleep, physical activity, nutrition, sexuality, contraception, pregnancy and maternity, alcohol risks, risks concerning tobacco and e-cigarette, cannabis and other synthetic drugs, stress, anxiety or depression, skin problems, vaccinations, environment and health risks, pain, and illnesses), why they had looked for Web-based health-related information per health topic (3 items: for yourself concerning a specific disease or medical problem which might affect you, out of curiosity, for your studies), and their main source of health information (7 items: forums, general health websites, YouTube, social networks such as Facebook and Twitter, institutional or official websites, blogs, and Wikipedia). They were also asked to rate the trustworthiness of each of these sources (3 items: credible, neither credible nor noncredible, and noncredible), and whether, from the beginning of their university studies, they had already looked online for a health professional or service (2 items: yes, no).
- Questions about opinions on digital health: participants were asked whether obtaining Web-based health information had resulted in a consultation with a health professional or service (2 items: yes, no), their reasons for consulting (3 items: information was insufficient, information was alarming, and information confirmed a real health problem) or not consulting (2 items: information was sufficient and information was not sufficient), and whether Web-based information and advice can be a complementary solution to real-life consultations (2 items: yes, no). Those answering positively to this question were further asked to report when searching for Web-based information could be most useful (3 items: before a consultation to get prepared, after a consultation to better understand the health professional’s instructions, and before and after a consultation). Those answering negatively were further asked to state whether Web-based information and advice could be an alternative to real-life consultations now or in the future (4 items: strongly agree, agree, disagree, and strongly disagree).

The English version of the questionnaire is available in Multimedia Appendix 2. The time of administration and completion of the questionnaire was about 10 min.

### Ethical Considerations

Ethics approval was obtained through the submission of a declaration detailing the survey implementation and questionnaire items to the attention of the French data protection authority, *Commission Nationale de l'Informatiqueet des Libertés* (National Commission of Informatics and Liberties). The written informed consent dated and signed by participants before answering the questionnaire reassured students of the anonymous format of the survey and use of collected data for research purposes only. For students who refused to participate in the study, we could collect paradata, that is, data documenting the process of data collection, such as reasons for refusal and information on campus. As a rule, paradata for each sampled person are completely anonymous and can be used for scientific purposes such as preventing or reducing high refusal rates without prior ethics approval [29].

### Statistical Analysis

Statistical analysis was performed using SAS (V.9.4; SAS Institute Inc, Cary, NC, USA). Descriptive statistics (eg, means and SDs) were used in the initial data analysis. Chi-square and Fisher exact tests were used to identify associations between sociodemographic characteristics, self-rated general and mental health, and digital health use of the study participants. For the tests of independence, digital health use was summarized in the following 5 components: (1) possessing a mobile health app, (2) possessing a health-related wearable device, (3) searched Web-based health-related information and support topics (for all reasons), (4) consulted Web-based sources for health-related information and support (for all degrees of credibility), and (5) searching online for a health professional or service. The level of statistical significance was set at *P* value <.05.

## Results

### Participants

A total of 777 students were approached to answer the survey: 591 of them participated in the study with a response rate of 76.0%. Students who refused to participate in the study were more frequently studying in Life and Health Sciences (71/186, 38.2%) and Law and Economy campuses (59/186, 31.7%). The majority of nonrespondents (99/186, 53.3%) declared they had no time or did not feel like answering a questionnaire. Reasons for refusal for remaining students (87/186, 46.7%) were that they had to attend a class, study at the campus library, or pass their examinations.

A total of 18 students were excluded because their date of birth was missing, 6 because they were younger than 18 years, and 59 because they were older than 24 years (according to the inclusion criteria). A student from a private higher education institute in Bordeaux was excluded as well. The final study sample included 507 students. Missing values were less than 12% and concerned mainly the following items: sources of Web-based health information (59/507, 11.6%), health-related information and support topics (31/507, 6.1%), and consulting or not a health professional or service after having obtained Web-based health information (15/274, 5.5%). We observed that missing values were more numerous for conditional questions and questions presented in a table format. The design of some items of our questionnaire may then explain nonresponse in our study. Missing values were excluded from both the descriptive analyses and the tests of independence.

The sociodemographic and health-related characteristics of study participants are summarized in Table 1.

The mean age of the whole sample was 20.5 years, 59.8% (303/507) of the participants were females, and 43.3% (220/507) were attending the first year of study, as shown in Table 1. As planned by design, the distribution of our sample did not differ from the distribution of the entire University of Bordeaux in 2016/2017 (data available in Multimedia Appendix 1) with regard to gender and field of study (*P*=.72). More than half of the participants rated both their general and mental health as good, 61.9% (314/507) and 57.6% (292/507), respectively. There were no missing values for data on sociodemographic characteristics and self-rated general and mental health.

### Questions About the Use of Digital Health

Concerning mobile-based digital health, almost all students (98.2%, 498/507) declared possessing a mobile phone, and among them, 34.9% (174/498) had at least 1 mobile health app, 62.6% (109/174) were using it occasionally, 27.6% (48/174) often, and 9.8% (17/174) never. Most mobile phone apps were about physical activity, for example, running, fitness (49.4%, 86/174), and general health monitoring (41.4%, 72/174). Other mobile health apps were about sleep (16.7%, 29/174), nutrition (8.0%, 14/74), wellness, for example, yoga (5.7%, 10/174), and gynecology (4.0%, 7/174, and, specifically among female students, 5.5%, 7/127). Moreover, 2 students reported having downloaded a mobile phone app for addictions, whereas 1 student for allergies. Some students (34.5%, 60/174) reported that they had not downloaded such apps, but that they were directly installed on their mobile phones, such as the Health iPhone app. Only 3.9% (20/507) of participants declared having a health-related wearable device.

**Figure 1:**
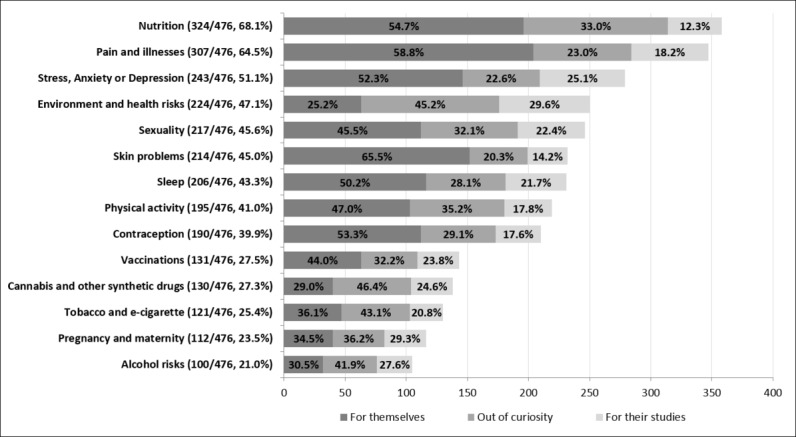
Health-related topics sought on the Internet and reasons.

Concerning internet-based digital health, 94.5% (450/476, with 31 missing values) of students had searched for Web-based information and support on at least 1 health-related topic in the last 12 months. The mean number of health-related topics students had searched for was 5.3 (SD 3.4). For each topic, students were asked to select one or more reasons for Web-based information and support seeking: 78.8% (375/476) mostly searched for themselves concerning a specific disease or medical problem which might affect them, whereas 61.6% (293/476) out of curiosity, and 39.9% (190/476) for their studies. Whatever the reason, the most searched topics were nutrition (68.1%, 324/476); pain and illnesses (64.5%, 307/476); and stress, anxiety, or depression (51.1%, 243/476). All results are shown in Figure 1.

**Figure 2:**
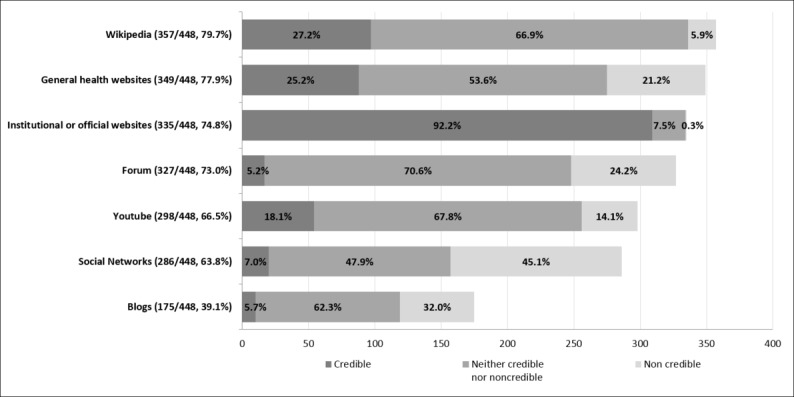
Web-based sources of health information and advice and their credibility.

Concerning Web-based sources of health-related information and advice, 99.1% (444/448, with 59 missing values) of students had consulted at least one of the proposed sources. The mean number of consulted Web-based sources was 4.5 (SD 1.9). While consulting several Web-based sources, students rated their credibility differently, as shown in Figure 2.

Although Wikipedia and general health websites were the most consulted sources (357/448, 79.7%, and 349/448 77.9%, respectively), students considered institutional or official websites as the most credible source (309/335, 92.2%,). Social networks and blogs were the least consulted sources (286/448, 63.8% and 175/448, 39.1%, respectively), and students rated them as the most noncredible sources of all (129/286, 45.1% and 56/175, 32.0%, respectively). Finally, 68.2% (344/504, with 3 missing values) of students had already looked online for a health professional or service from the beginning of their university studies.

**Table 1:** Sociodemographic and health-related characteristics of study participants (N=507).

Sociodemographic characteristics		n (%)
**Gender**		
	Female	303 (59.8)
	Male	204 (40.2)
**Field of study**		
	Literature and Social Sciences	91 (17.9)
	Life and Health Sciences	181 (35.7)
	Science and Technology	89 (17.6)
	Law and Economy	146 (28.8)
**Year of study**		
	1st year	220 (43.3)
	2nd year	112 (22.1)
	3rd year	91 (17.9)
	>3rd year	84 (16.7)
**Self-rated general health**		
	Very good	65 (12.8)
	Good	314 (61.9)
	Average	112 (22.1)
	Bad	15 (3.0)
	Very bad	1 (0.2)
**Self-rated mental health**		
	Very good	94 (18.5)
	Good	292 (57.6)
	Average	100 (19.7)
	Bad	17 (3.4)
	Very bad	4 (0.8)

### Sociodemographic and Self-Rated General and Mental Health Correlates of Digital Health Use

We examined the correlation of digital health use, defined by 5 components, with sociodemographic characteristics and self-rated general and mental health. Table 2 reports the detailed results. Gender was significantly associated with all components of digital health use. More precisely, female students were almost twice as likely to use a mobile health app compared with male students (*P*<.001). Inversely, male students were more than twice as likely to have a health-related wearable device compared with female students (*P*=.04). However, when interpreting this result, it is important to consider the small number of subjects possessing a health-related wearable device (n=17). Female students used the internet for health information and support seeking as well for searching a health professional or service significantly more than male students (*P*<.001 and *P*=.002, respectively). The field of study was significantly associated with possessing a health-related mobile phone app (*P*=.03), searching the internet for health-related information and support topics (*P*=.001), and looking online for a health professional or service (*P*<.001). For these 3 components of digital health use, the highest proportions of students were found in Literature and Social Sciences, as well as in Life and Health Sciences. The year of study was significantly associated with searching online for a health professional or service (*P*<.001). No statistically significant association was found between all components of digital health use and both self-rated general and mental health status. However, as for health-related wearable devices, the number of students rating both their general and mental health as bad or very bad was small, and results should be interpreted with caution.

**Table 2:** Sociodemographic and self-rated health correlates of digital health use. All values are given excluding missing values for each separate component.

Variable		Mobile health app (N=498), n (%)	Health-related wearable device (N=504), n (%)	Health-related information and support topics (N=476)		Consulted online sources (N=448)		Searching health professional or service online (N=504), n (%)
				n	Mean (SD)	n	Mean (SD)	
Overall		174 (34.9)	17 (3.4)	476	5.3 (3.4)	448	4.5 (1.9)	344 (68.3)
**Gender**		*P<*.001	*P=*.04	*P<*.001		*P=*.049		*P=*.002
	Female	127 (42.8)	6 (2.0)	283	5.8 (3.3)	278	4.3 (1.8)	222 (73.5)
	Male	47 (23.4)	11 (5.4)	193	4.7 (3.4)	170	4.7 (1.9)	122 (60.4)
**Field of study**		*P=*.03	*P*=.07	*P=*.001		*P=*.52		*P<*.001
	Literature and Social Sciences	35 (39.8)	1 (1.1)	86	6.4 (3.3)	85	4.2 (2.0)	68 (75.6)
	Life and Health Sciences	71 (40.1)	11 (6.1)	174	5.6 (3.8)	155	4.6 (1.7)	138 (76.2)
	Science and Technology	20 (22.7)	3 (3.4)	84	4.4 (2.9)	77	4.4 (1.8)	49 (55.7)
	Law and Economy	48 (33.1)	2 (1.4)	132	4.9 (2.9)	131	4.5 (1.9)	89 (61.4)
**Year of study**		*P=*.89	*P=*.80	*P=*.86		*P=*.94		*P<*.001
	1st year	79 (36.2)	7 (3.2)	206	5.3 (3.4)	186	4.5 (1.8)	128 (58.4)
	2nd year	37 (34.9)	4 (3.7)	103	5.1 (3.2)	94	4.4 (1.8)	67 (63.2)
	3rd year	32 (35.2)	2 (2.2)	87	5.4 (3.2)	91	4.5 (1.9)	73 (78.5)
	>3rd year	26 (31.3)	4 (4.7)	80	5.7 (3.8)	77	4.4 (1.9)	76 (88.4)
**Self-rated general health**		*P=*.31	*P=*.11	*P=*.39		*P=*.21		*P=*.42
	Very good	16 (25.4)	3 (4.6)	62	4.8 (3.7)	55	4.0 (1.7)	38 (58.5)
	Good	118 (37.9)	14 (4.5)	293	5.5 (3.4)	278	4.5 (1.9)	218 (69.9)
	Average	36 (33.0)	0 (0.0)	107	5.3 (3.1)	99	4.5 (1.8)	77 (69.4)
	Bad	4 (28.6)	0 (0.0)	13	4.8 (3.1)	15	4.2 (1.6)	10 (66.7)
	Very bad	0 (0.0)	0 (0.0)	1	2.0 (N/A^a^)	1	5.0 (1.6)	1 (100.0)
**Self-rated mental health**		*P=*.95	*P=*.08	*P=*.30		*P=*.66		*P=*.49
	Very good	34 (36.6)	6 (6.5)	91	5.1 (3.6)	81	4.6 (1.7)	62 (66.7)
	Good	102 (35.7)	7 (2.4)	271	5.2 (3.4)	261	4.4 (1.9)	199 (68.6)
	Average	31 (31.6)	2 (2.0)	96	5.8 (3.1)	89	4.5 (1.8)	65 (65.0)
	Bad	6 (35.3)	2 (11.8)	14	5.7 (3.6)	13	3.8 (2.1)	14 (82.4)
	Very bad	1 (25.0)	0 (0.0)	4	4.8 (2.6)	4	5.0 (1.6)	4 (100.0)

^a^N/A: not applicable.

With regard to the second component of digital health use, we further distinguished the reasons for searching health-related information and support topics online and found that self-rated mental health was significantly associated with a higher mean number of health-related topics searched for themselves, concerning a specific disease or medical problem which might affect the respondents (*P*<.001). We observed a mean of 2.4 (SD 2.5) topics for students reporting very good mental health and a mean of 3.8 (SD 2.6) topics for students rating their mental health as bad. Concerning the field of study, the association with the online search for health-related topics remained significant for the specific reasons, for themselves, and for their studies (*P*<.001 both). However, for each separate reason, the mean number of searched topics was different across the fields of study. On the one hand, the mean number of searched health-related topics “for themselves” was higher in Literature and Social Sciences (4.0, SD 2.9) than in the other fields of study (the lowest mean number being 2.1, SD 1.8, in Law and Economy). On the other hand, the mean number of searched health-related topics for their studies was largely higher in Life and Health Sciences (2.5, SD 3.1) than in the other fields of study (the lowest mean number being 0.3, SD 0.6, in Law and Economy).

### Questions About Opinions on Digital Health

Students who reported having searched for at least 1 health-related topic online (N=450) were asked whether information found online had induced them (or not) to consult a health professional or service, as well as related reasons. A total of 38.8% (174/448, with 2 missing values) declared that information found online had induced them to access care. Reasons were that online information had confirmed a real health problem (50.6%, 88/174), online information was insufficient (37.9%, 66/174), and online information was alarming (30.5%, 53/174). On the contrary, 61.2% (274/448) of students declared that information found online had not induced them to access care. Reasons were that online information was sufficient (78.4%, 203/259, with 15 missing) and online information was reassuring (31.7%, 82/259).

A total of 49.7% of students (251/505, with 2 missing values) declared that online information and advice can be a complementary solution to real-life consultations, before a consultation to get prepared (50.4%, 126/250, with 1 missing), before and after a consultation (32.4%, 91/250), and after a consultation to better understand the health professional’s instructions (17.2%, 43/250).

Finally, majority of the students reported that they “strongly disagreed” or “disagreed” that today’s digital health can replace real-life consultations, 55.5% (280/505, with 2 missing values) and 38.6% (195/505), respectively. However, among them (n=464, with 11 missing values), 44.6% (207/464) reported that, in the near future, digital health would replace real-life consultations but only if promoted by institutional or official entities, for example, the national ministry of health and the university.

## Discussion

### Digital Health Use and Correlates

We described digital health use among university students as a multidimensional concept given by 5 components. Regarding the first component (possessing a mobile health app), our results confirmed the large penetration of mobile phone ownership among young people, with almost all participants (498/507, 98.2%) possessing a mobile phone, in line with national statistics (89% of students had a mobile phone in France in 2015) [30] and international ones (more than 80% of people aged 18-34 years had a mobile phone in OECD countries in 2015) [31]. However, in our sample, the use of mobile health apps was less spread: only one-third of students had a mobile health app and used it mostly occasionally. We can hypothesize that university students do not use largely mobile health apps because of the demanding nature of data entry [32], as well as limited storage memory and battery life of their mobile phone [33]. Survey metrics about the use of mobile health apps in the student population worldwide are scarcely documented. A few studies have been conducted in US college students, focusing on fitness and wellness apps [34,35], whereas some qualitative studies have explored the views and experiences of European students on mobile phone apps related to health behavior change [36,37]. Results from our survey and previous studies confirm that students’ most-used mobile health apps concern physical activity (eg, running, fitness) and general health monitoring, such as the Health iPhone app. These findings can be interpreted in 2 opposite ways. First, they might suggest that future effective and successful digital health interventions should be based on mobile phone apps for physical activity and general health monitoring because it is well assessed that students appreciate and use them. Second, the opposite interpretation suggests that, because such apps already exist, future digital health interventions should be based on mobile phone apps concerning different health topics, to help students take care of other aspects of their health and well-being. Some mobile phone apps on addictions, sexual risks, and mental health have been developed, tested, and validated among students [13-16] and could be largely disseminated to the general student population. Future research should monitor the diffusion, use, and acceptability of such apps, investigating reasons for (non)adoption and (non)continuance of use.

Concerning the second component (possessing a health-related wearable device), only 1 student out of 25 owned a wearable device for health purposes. However, recent surveys on the general population showed that young people aged between 18 and 35 years represent the highest consumers of wearable devices, ranging from 36% to 49% of the overall interviewed populations [38,39]. Furthermore, a study carried out in the Cardiff Metropolitan University [40] reported that 35% (18/51) of interviewed students aged 18-30 years owned a wearable device. For the remaining 65% (33/51) of students, the main reasons for not owning such a device were concerns about electromagnetic waves emitted by wearable devices, security risks concerning collected data, reluctance to wear the device continuously, and costs which are not always affordable. Our low percentage of students owning a wearable device might be because of one or more of these reasons.

With regard to the third component (searching for health-related information and support online), the level of use of the internet for health-related information seeking for personal reasons reported in our sample (375/476, 78.8%) was slightly higher than prevalence estimates (ranging from 66.1% to 67.7%) found in other university-based surveys worldwide [18,19,27]. We also looked at other reasons for health-related information and support seeking among university students, including for curiosity and for one’s studies. All reasons considered, the level of use of the internet for health-related information seeking found in our sample was very high (94.8%, 450/476). These high percentages suggest that the internet represents a very attractive platform to deliver a digital health intervention targeting students. Given the lower use of mobile phone apps compared with the high use of the internet for health purposes, our results suggest that future digital health interventions should be based on mobile-responsive design websites rather than on mobile apps. Web apps could be the most cost- and time-efficient delivery solution for this specific target group. We also observed that the most searched topics in our sample were the same as those reported in previous studies [18,19,27], with pain, illnesses, and nutrition being the most popular health-related topics among surveyed students. On the basis of these findings, future digital health interventions could address these topics to meet students’ interests and needs.

As for the fourth component (consulted online sources), almost all students (444/448, 99.1%) had consulted 1 or more online sources to get health-related information and support. Even if Wikipedia and general health websites were the most consulted sources, university students rated institutional or official websites as the most credible source. This suggests that university students show discerning judgment and pay attention to the trust and credibility of the websites and platforms they consult [41]. Our findings are in line with previous research, reporting that authority of the sources and disclosure of the authors are among the main criteria students use for assessing the accuracy of the information found online [19]. Digital health interventions proposed within the university setting by recognized authorities (eg, health professionals, and faculty) have huge potential in this specific population.

Finally, with regard to the fifth component (searching online for a health professional or service), we observed that one-third of students had already used the internet to search and contact a health professional and service. This might be explained by the fact that students often live far from their family and hometown and recur to the internet to find a health professional or service near their new accommodation. Digital health interventions displaying the closest, safest, and most appropriate health services could meet the needs of a good portion of university students [23].

Effective engagement in a digital health intervention requires careful consideration of current digital health use, but also of personal factors such as sociodemographic characteristics and health status. For this reason, we investigated correlates of digital health use in our sample. Gender was significantly associated with all components of digital health use. Female students were more likely to use mobile health apps and to use the internet for health information and support as well as for searching a health service or professional. Male students, instead, consulted more online sources and possessed more wearable devices compared with female students. These findings are in line with research reporting that women are more engaged in using the internet for health-related information searching because of their higher health awareness and personal disposition of being well-informed as potential patients [42,43]. On the other hand, the higher number of consulted online sources and wearable devices among male students could be because of the fact that men ascribe themselves higher perceived digital and technological competencies [42].

As for the year of study, we were interested in exploring whether freshmen were using digital health differently from other students. The freshmen year of university is a critical period where many social and environmental factors act on students influencing their well-being and putting their health at risk [44]. We did not find any strong association between the year of study and digital health use, but future research should focus on first-year students who usually struggle to cope with their transition to university.

We also expected that university students’ digital health use would differ across fields of study, and that, more precisely, students in Life and Health Sciences would use digital health more than their colleagues from other disciplines, given their personal and study interests. Our hypothesis was confirmed because students in Life and Health Sciences were the highest digital health users in our sample. However, students in Literature and Social Sciences, as well as in Law and Economy, were also largely using digital health, especially for personal reasons. Digital health use in Life and Health Sciences can be easily justified by the fact that medical and health students need to be knowledgeable about online health information resources and to stay up-to-date with digital health tools for their studies as well as for their future career as health professionals. Besides, in France, some university curricula are highly demanding and stressful, such as Medicine and Law. Digital health interventions carried out in the university setting should take into account differences across fields of study, targeting students who might be at higher risk of mental health distress, for instance.

Among all personal factors, health is a very important part of the field of consumer health [45]. In our study, neither self-rated general nor mental health was correlated with any component of digital health use. Even if these results must be interpreted with caution because the number of subjects rating their health as bad or very bad was small, thus limiting the strength of our analysis, it is interesting to observe that students were active digital health users independently from their self-rated health status. Practically, this implies that digital health interventions should not be limited exclusively to treatment and care, but could be very useful for preventing diseases and promoting health. University students are generally in good health, as confirmed by our findings, but digital health can help improve and maintain health consciousness in this population [46].

### Opinions on Digital Health

We also explored whether seeking Web-based health information influenced students’ consultations with health professionals. We found that more than half of the students did not consult any health professional after obtaining Web-based health information, mostly because the information was sufficient. On the contrary, for students having consulted a health professional after obtaining Web-based health information, the main reason was that the obtained information had confirmed they had a real health problem to treat. These findings could suggest that health information obtained on the internet can motivate young people to have a consultation with a health professional, but only if they think they have a real or rather serious problem to take care of. In this transitional phase where students are moving toward attaining autonomy and assuming responsibility for their health care [47], Web-based health-related information can represent support. However, future qualitative studies are warranted to better explore how digital health influences the health-seeking behavior of students.

A prevailing view among participants of our study was that digital health should be an adjunct rather than a replacement to real-life consultations. Digital health was considered most impactful as a mean of enhancing health care services, before or after consultations. Importantly, when asked about the future of digital health, the subset of students who disagreed with the statement regarding Web-based information or advice being an alternative to real-life consultations was positive that internet- and mobile-based health tools could have the potential to replace real-life consultations, provided that such tools are promoted by institutional or official entities, for example, the national ministry of health. Institutions continue to play a central role in most students’ lives, especially when it comes to obtaining health information, being treated, and maintaining good health [48]. Therefore, promoting digital health interventions in a university setting seems to be a promising approach because health and academic authorities are considered as a trustful source of health-related messages and advice.

### Limitations

Our study relied on data by a middle-size sample of students, resulting into a small number of units of analysis in some variable categories (eg, self-rated general and mental health). This might have reduced the power of our study and increased margin of error concerning the estimated associations. The field survey methodology may represent a further limitation: questionnaires were administered on campuses during courses and examinations. Interviewers may have been biased in who they decided to approach based on walking speed, what students looked like, or whether they were waiting before a class, for instance. Furthermore, participants might have been not completely at ease when answering the questionnaire because of their timetable, stress for examinations, academic workload, and so on. The face-to-face administration of the questionnaire may represent another bias. Participants might have not felt free to disclose to their peer interviewers that they were concerned by some health problems or that they were interested in specific sensitive health topics such as depression, sexuality, or addictions. Although this bias must be carefully taken into account, it is also noteworthy that, after questionnaire completion, some participants reported to their peer interviewers that they were content with the fact that university researchers were investigating about their health and well-being. The peer-to-peer approach was chosen to maximize the comfort of participants. Students were reassured by their peer interviewers on the possibility to interrupt the survey if they considered it too intrusive and on the fact that university researchers conducting the analyses would not be able to recognize any participant. Finally, our questionnaire did not use validated measures or scales but was constructed by combining items from previous surveys in university students with new ad hoc questions covering our topics of interest. However, both the test phase and the following survey implementation proved that the questionnaire was easy to administer and participants answered readily. Further research, both qualitative and quantitative in nature, including a larger and more representative sample, would improve the findings by describing university students’ reasons to use digital health, their behavioral goals, and intention to continuously use digital health. The definition of digital health use could also be enlarged in future studies by exploring more deeply social media use, for instance, as well as other components of the use of both internet- and mobile-based tools for health, such as telehealth technologies and electronic health records [49].

### Implications

Although the generalizability of our findings is limited by being based on a sample of university students from one country, our study can provide the wider international community with useful information on how to plan and implement future digital health interventions in the university setting. First, we cataloged the health topics of interest for university students, suggesting some contents for new digital health interventions. Second, we confirmed that university students demand for high-quality health-related information and support, especially in the digital environment. Third, our findings suggested that university students are mostly using the Web (internet and social media), rather than mobile phone apps and wearable devices: at present, bracelets or smartwatches are not the first options for implementing a digital health intervention addressing university students.

Finally, the questionnaire we proposed could be improved and applied in other universities before the conception, development, and diffusion of digital health interventions. Conducting a survey to collect baseline data on university students’ needs and opinions with regards to digital health can provide an initial macro-level evidence base that can be used to guide the university’s digital health strategy. Similar survey studies, also combined with in-depth qualitative studies, would allow university staff (eg, faculty and health professionals in student health centers) to get more insights on how to design effective digital health interventions (eg, choice of the most appropriate e-tool, topics of interest) and how to diffuse them according to different students’ profiles.

### Conclusions

In an exploratory approach, we provided a picture of current use and opinions about digital health among university students in France to shed some light on the conception, development, and diffusion of future digital health interventions addressed to this specific public. With the internet still outpacing mobile health apps and wearable devices as sources of health information and support among university students, this population is confident that digital health interventions will replace real-life consultations in the future, provided that they are promoted by official institutions such as the university or the national ministry of health.

## Appendix

Multimedia Appendix 1 Table reporting the distribution of students at the University of Bordeaux and in our study according to gender and field of study.

Multimedia Appendix 2 English version of the questionnaire.

## Abbreviations

OECD: Organisation for Economic Co-operation and Development
