# Supplementary material for: Exploring Digital Health Use and Opinions of University Students: Field Survey Study
Source: JMIR Mhealth Uhealth. 2018 Mar 15;6(3):e65. doi: 10.2196/mhealth.9131 (PMC5876492; doi:10.2196/mhealth.9131)
Supplement: Multimedia Appendix 1 [file mhealth_v6i3e65_app1.pdf]

MULTIMEDIA APPENDIX 1

Table A. Distribution of students at the University of Bordeaux (N=70,000) and in our study (N=507) according to gender and field of study

	University of Bordeaux						Our Study					
	Female		Male		Total		Female		Male		Total	
**Law and Economy**	12,833	(55.6%)	9,333	(44.4%)	22,167	(31.6%)	85	(58.2%)	61	(41.8%)	146	(28.8%)
**Life and Health Sciences**	15,167	(65.0%)	8,167	(35.0%)	23,333	(33.4%)	115	(63.5%)	66	(36.5%)	181	(35.7%)
**Literature and Social Sciences**	8,167	(70.0%)	3,500	(30.0%)	11,667	(16.7%)	69	(75.8%)	22	(24.2%)	91	(17.9%)
**Science and Technology**	4,667	(36.4%)	8,167	(63.6%)	12,833	(18.3%)	33	(37.1%)	56	(62.9%)	89	(17.6%)
**Total**	**40,833**	**(58.3%)**	**29,167**	**(41.7%)**	**70,000**	**(100%)**	**302**	**(59.5%)**	**205**	**(40.5%)**	**507**	**(100%)**

Female students of our sample were 302 (59.5%) vs 40,833 in the University of Bordeaux (58.3%); students in Literature and Social Sciences were 91 (17.9%) vs 11,667 (16.7%); students in Life and Health Sciences were 181 (35.7%) vs 23,333 (33.4%); students in Science and Technology were 89 (17.6%) vs 12,833 (18.3%); students in Law and Economy were 146 (28.8%) vs 22,167 (31.6%).
