# Supplementary material for: Exploring Digital Health Use and Opinions of University Students: Field Survey Study
Source: JMIR Mhealth Uhealth. 2018 Mar 15;6(3):e65. doi: 10.2196/mhealth.9131 (PMC5876492; doi:10.2196/mhealth.9131)
Supplement: Multimedia Appendix 2 [file mhealth_v6i3e65_app2.pdf]

MULTIMEDIA APPENDIX 2 – ENGLISH VERSION OF THE QUESTIONNAIRE

Questionnaire Code

Date

Nome

Campus

Your use and opinions of digital health

SECTION 1: Who are you?

1. **Gender**: Male/Female
2. **Which are your month and year of birth?**
3. **Which is your field of study?**

☐ Science and Technology

☐ Literature and Social Sciences

☐ Law and Economy

☐ Life and Health Sciences

4. **Which is your current year of study?**

☐ 1st year ☐ 2nd year ☐ 3rd year ☐ > 3rd year

5. **How would you rate your general health status?**

☐ Very good

☐ Good

☐ Average

☐ Bad

☐ Very bad

6. **And what about your mental health?**

☐ Very good

☐ Good

☐ Average

☐ Bad

☐ Very bad

SECTION 2: Use of digital health

7. **Do you have a smartphone?**

☐ Yes

☐ No (*Go to question 11*)

8. **Do you have a health-related application (nutrition, running, sleep, well-being…) ?**

☐ Yes

☐ No (*Go to question 11*)

9. **If yes, please provide the name**

10. **Which is your frequency of use?**

☐ Often

☐ Occasionally

☐ Never

11. **Do you have a health-related wearable device like a bracelet or a smartwatch?**

☐ Yes

☐ No

12. **During the last 12 months, have you looked for online information or support on these health and well-being topics?** *(MULTIPLE CHOICE)*

Topics	***YES***			***NO***
	For yourself concerning a specific disease or medical problem which might affect you	***Out of curiosity***	***For your studies***	
Sleep				
Physical activity and its repercussion on health				
Nutrition (weight, health habits, eating disorders, food composition)				
Sexuality (sexual habits, homosexuality, sexually transmitted diseases, HIV)				
Contraception (condom, pill, IUD, implant)				
Pregnancy and/or maternity				
Alcohol risks				
Risks concerning tobacco and e-cigarettes				
Cannabis and other synthetic drugs				
**Stress/anxiety/depression**				
Skin problems				
Vaccinations				
Environment and health risks				
Pain and illnesses				
Other topics:				

** If a student answers NO to all items, do not administer the questions underlined in grey.*

13. **What are the sources of information you explored? How do you rate the information found?**

	YES			NO
	**Credible information**	**Neither credible or non-credible information**	**Non-credible information**	
Forums				
General health websites				
YouTube				
Social networks (Facebook, Twitter)				
Institutional/official websites				
Blogs				
Wikipedia				

14. Since the beginning of your university studies have you already looked online for a health professional/service (ex. general practitioner, dentist, gynaecologist)?

☐ Yes

☐ No

SECTION 3: Opinions on digital health

15. **You have declared that you have already looked for health-related information online for a personal problem. Has this resulted in a consultation with a health professional/service?**

☐ Yes

☐ No

15.1. If YES, why? *(MULTIPLE CHOICE)*

☐ Information was insufficient

☐ Information was alarming

☐ Information confirmed a real health problem

15.2. If NO, why? *(MULTIPLE CHOICE)*

☐ Information was sufficient

☐ Information was reassuring

16. **Do you think online information and advice can be a complementary solution to real-life consultations?**

☐ Yes

☐ No

16.1. If YES, when could they be most useful?

☐ Before a consultation to get prepared

☐ After a consultation to better understand the health professional's instructions

☐ Before and after a consultation

17. **Do you think online information and advice could be an alternative to real-life consultations now or in the future?**

☐ Strongly agree

☐ Agree

☐ Disagree

☐ Strongly disagree

17.1. If DISAGREE/STRONGLY DISAGREE, do you think this will be possible in the future (for instance through a validated website by the ministry of health)?

☐ Yes

☐ No
